# ARE SOCIAL VULNERABILITY INDEX AND COGNITIVE DECLINE RELATED?

**DOI:** 10.1093/geroni/igae098.1558

**Published:** 2024-12-31

**Authors:** Brandy Sutphin, Sharada Sarah Adolph, Bala Simon

**Affiliations:** Arkansas Department of Health, Little Rock, Arkansas, United States; Arkansas Department of Health, Little Rock, Arkansas, United States; Arkansas Department of Health, Little Rock, Arkansas, United States

## Abstract

Alzheimer’s disease and related dementias (ADRD) prevalence is demonstrably increasing as more people live to advanced age. ADRD was Arkansas’s 7th leading cause of death in 2021 and an estimated 67,000 Arkansans will be living with ADRD by 2025. While known ADRD risk factors hypertension, obesity, smoking, heavy metal exposure, and familial inheritance are well studied, there is minimal research examining the influence of social vulnerability on ADRD patients. We sought to study population-level associations between the Centers for Disease Control and Prevention (CDC), Agency for Toxic Substances and Disease Registry (ATSDR) Social Vulnerability Index (SVI) and cognitive decline, the earliest symptom of ADRD, to identify high-risk areas for interventions. An ecological study design at county-level was utilized. CDC/ATSDR SVI scores <0.5 indicate low-to-medium risk and scores ≥0.5 indicate medium-high-to-high risk of social vulnerability. Associations were tested between SVI composite and four component themed indices as predictor variables and Behavioral Risk Factor Surveillance System (BRFSS) cognitive decline prevalence as the outcome variable. Arkansas has 26 high-risk counties out of 75 (34.7%) with combined SVI ≥0.5 and high cognitive decline prevalence (µ ≥12.1%). Regression showed unit increases in SVI ≥0.5 resulted in significant increases in cognitive decline prevalence for composite SVI (β=7.67, p=0.0185), and corresponding component SVIs socioeconomic status (β=6.75, p=0.0494), household characteristics (β=9.28, p=0.0032), and racial and ethnic minority status (β=8.98, p=0.0458). Significant ecological associations indicate potential reciprocal influences between SVI and cognitive decline and the need for targeted interventions in high-risk counties.

